# Completeness of birth and death registration in a rural area of South Africa: the Agincourt health and demographic surveillance, 1992–2014

**DOI:** 10.3402/gha.v9.32795

**Published:** 2016-10-24

**Authors:** Michel Garenne, Mark A. Collinson, Chodziwadziwa W. Kabudula, F. Xavier Gómez-Olivé, Kathleen Kahn, Stephen Tollman

**Affiliations:** 1 MRC/Wits Rural Public Health and Health Transitions Research Unit, School of Public Health, Faculty of Health Sciences, University of the Witwatersrand, Johannesburg, South Africa; 2 Institut de Recherche pour le Développement, UMI Résiliences, Bondy, France; 3 Institut Pasteur, Epidémiologie des Maladies Emergentes, Paris, France; 4 FERDI, Université d'Auvergne, Clermont-Ferrand, France; 5 INDEPTH Network, Accra, Ghana; 6 Umeå Centre for Global Health Research, Division of Epidemiology and Global Health, Department of Public Health and Clinical Medicine, Umeå University, Umeå, Sweden

**Keywords:** completeness of vital registration, demographic surveillance system, socioeconomic factors, level of education, household wealth, Mozambican refugee, South Africa, Agincourt

## Abstract

**Background:**

Completeness of vital registration remains very low in sub-Saharan Africa, especially in rural areas.

**Objectives:**

To investigate trends and factors in completeness of birth and death registration in Agincourt, a rural area of South Africa covering a population of about 110,000 persons, under demographic surveillance since 1992. The population belongs to the Shangaan ethnic group and hosts a sizeable community of Mozambican refugees.

**Design:**

Statistical analysis of birth and death registration over time in a 22-year perspective (1992–2014). Over this period, major efforts were made by the government of South Africa to improve vital registration. Factors associated with completeness of registration were investigated using univariate and multivariate analysis.

**Results:**

Birth registration was very incomplete at onset (7.8% in 1992) and reached high values at end point (90.5% in 2014). Likewise, death registration was low at onset (51.4% in 1992), also reaching high values at end point (97.1% in 2014). For births, the main factors were mother's age (much lower completeness among births to adolescent mothers), refugee status, and household wealth. For deaths, the major factors were age at death (lower completeness among under-five children), refugee status, and household wealth. Completeness increased for all demographic and socioeconomic categories studied and is likely to approach 100% in the future if trends continue at this speed.

**Conclusion:**

Reaching high values in the completeness of birth and death registration was achieved by excellent organization of the civil registration and vital statistics, a variety of financial incentives, strong involvement of health personnel, and wide-scale information and advocacy campaigns by the South African government.

## Introduction

The registration of vital events (births and deaths) is a crucial element of modern life, as it determines many rights and duties in modern societies and is necessary for administration and development planning. Vital registration is also an important source of data for demographic and public health research. In fact, many of the early investigations of population and health dynamics (fertility and mortality) and patterns (age and sex) were based on long-term time series of vital statistics. Compulsory registration of births and deaths has a long history in Europe, going back to the Middle Ages through parish registers and since the eighteenth century for civil registration (1, 2). Vital registration has been complete since the early nineteenth century in Western Europe and since the early twentieth century in most industrialized countries (North America, Australia, New Zealand, Japan, Russia, etc.). It is also nearly complete in many Latin American countries and in selected Asian countries but remains incomplete in many other developing countries. Thanks to numerous initiatives from a variety of institutions (UNFPA, bilateral aid agencies, the African Union, Bloomberg, etc.), many projects were developed in the past decade to improve civil registration and vital statistics (CRVS) in areas where completeness is still low (3–10).

Africa is a special case with respect to vital registration (11–15). Only a few African countries have maintained a complete or near-complete vital registration for a long time. This is particularly the case for islands (Mauritius, Reunion, Seychelles, and Sao Tome and Principe) and to a certain extent for some North African countries (Egypt). In sub-Saharan Africa, there are examples of near-complete vital registration in selected populations, in particular in capital cities (Dakar, Brazzaville, Antananarivo) and among selected groups (such as white Europeans in South Africa). Otherwise, the registration of births and deaths has remained very low for most African populations, despite many laws passed and many attempts to improve registration (16–20). However, this situation is changing rapidly in Southern Africa, where major efforts have taken place in recent years to reach near-complete registration of births and deaths. This is the case in South Africa, as well as in nearby Namibia and Botswana, who embarked on large programs to improve vital registration (15).

There is a long history of vital registration in South Africa but with serious discrepancies by population group and geographical area. The first law for compulsory birth and death registration was passed in 1867, but it affected primarily the white European population of the Cape Colony. The 1923 Births, Deaths and Marriages Registration Act made compulsory the registration of vital events for all persons living in urban areas, registration being left voluntary for those living in rural areas. During the apartheid years (1948–1991), vital registration in the homelands was left to the homeland administrations and remained very deficient in these areas. The situation changed with the dismantlement of petty apartheid in 1986, and with the 1992 Births and Deaths Registration Act, the registration of all births and deaths to South African citizens and permanent residents became compulsory. These legal events, together with the strong will of the post-apartheid government and the reorganization of the civil registration system, radically changed the situation of vital registration in the country (21–26).

Estimating the completeness of birth and death registration in South Africa is difficult for several reasons. First, the denominator, that is, the precise number of total births and deaths that occur in the country, is controversial, and various estimates made from censuses, demographic surveys, and models vary by a margin of 10% or more. For instance, the number of births in 2001 was estimated at 1.088 million by the United Nations Population Division (UNPD), and estimates used by the South African Statistical Office (Stats-SA) ranged from 1.076 to 1.171 million, a 9% difference between high and low values. Estimates made from census data show even larger variations, from 0.947 million (2001 census) to 0.955 million (2007 community survey) and 0.971 million (2011 census), up to 13% lower than UNPD estimates. Second, the numerator, that is, the number of events effectively registered, may also be a source of confusion. For births in particular, there is a huge gap between births declared within 1 month (as by law), births declared during the same calendar year (called ‘current registration’), as published by the statistical office, and births declared later (called ‘late registration’), which may occur several years after the event. For instance, the number of 1992 births registered that same year was 228,445, but by 2015 it had become 981,258 because of late registration (4.1 times more!). Despite this double uncertainty, there is no doubt that the completeness of birth and death registration has improved markedly since 1992, above all for the black African populations, who were largely overlooked by vital statistics before 1991. Compared with UNPD estimates of births and deaths, birth registration (same year) increased from 21.2% in 1992 to 84.1% in 2012, while death registration increased from 50.4 to 71.2% over the same period of time. It should be noted that UNPD estimates of the number of deaths after 2005 are likely to be overestimated, therefore underestimating the completeness of death registration after this date. Stats-SA estimates of the completeness of birth registration showed an increase from 24.7% in 1998 to 72.0% in 2005 (23). Using the actuarial society of south africa (ASSA) model developed by Prof. Dorrington and colleagues as a reference, estimates of the completeness of death registration show an increase from 85 to 90% for adults and from 44 to 78% for children between 1996 and 2000 (25).

The aims of this study were to document trends in completeness of the registration of vital events and to investigate their sociodemographic factors in Agincourt, a rural area of South Africa under demographic surveillance. In this area, both numerators and denominators have been known accurately since 1992, and the health and demographic surveillance system (HDSS) provides numerous demographic and socioeconomic correlates at household and at individual level to investigate risk factors.

## Data and method

### Study area

The Agincourt HDSS has been described in detail elsewhere (27–31). It is located in the dry lowveld of the northeastern part of South Africa, near the Mozambican border. The area is now part of Mpumalanga Province and was formerly included in the Gazankulu and Lebowa homelands. It is a relatively poor rural area, populated mainly by the Shangaan ethnic group. It includes a sizeable community of Mozambicans from the same ethnic group, who came in the 1980s as refugees during the civil war and settled in South Africa; they now account for about 30% of the population in the HDSS area. The Mozambican refugees became better and better integrated over the years in terms of income, education, and demographic behavior, but they still have distinctive features, as shown in this study (32–35).

The HDSS area varied somewhat over the years. When it started in 1992, it included some 20 villages and a population of about 57,600 persons, and over the years, it integrated three newly created villages, part of the Reconstruction and Development Programme, which are mostly offshoots of former villages. The HDSS was extended to include four new neighboring villages in 2007, and another five villages between 2010 and 2012. The population counted some 110,000 inhabitants in total in 2015.

The HDSS is primarily a full population register, including the routine registration of births, deaths, in- and out-migration, and a variety of other events recorded during yearly home visits. The population register is updated by trained field staff, who interview the household with a prepopulated form based on information given the year before. This information includes the full household roster and the last-born child for each woman, which ensures complete registration of births and deaths. The HDSS is also the platform for numerous surveys and intervention trials. With respect to this study, births and deaths are recorded on questionnaires that include a special question on whether the birth or the death was registered. Because fieldworkers visit each household routinely once a year, the question on birth and death registration covers, on average, a time lag of 6 months, ranging from the date of the event to a year apart. As a result, the completeness of birth and death registration covers most cases of ‘current registration’ of vital events but not the so-called late registrations that were previously so prevalent in South Africa.

### Data and methods

The data used for the study cover all vital events (births and deaths) that occurred in the resident population over a 22-year period, from the first census conducted in 1992 to the last round conducted in 2014. Within the surveillance population, undercounting of births and deaths was negligible. The few births or deaths that might be not recorded include those occurring among very recent migrants, and newborns that end up as neonatal deaths. In particular, during the peak of the HIV epidemic, some people were coming back to die in their village of origin, some of which might have not been recorded (36). Likewise, some very young women might have gone to their family to deliver a baby while living elsewhere. In principle, only births and deaths that occurred within the resident population, duly recorded in the HDSS, were counted, as is normally done elsewhere in demographic surveillance systems. As a consequence, the births and deaths recorded by the HDSS might differ somewhat from those recorded at the local registration sites. The main advantage of the HDSS is the congruence between the numerator and denominator, necessary for a proper estimation on completeness.

The Agincourt population is homogeneous in terms of ethnicity, but is stratified by socioeconomic status. There are major differences in most demographic indicators by level of education and by level of household wealth, and one peculiarity of this population is the status of the Mozambican refugees. Level of education is routinely recorded and updated in censuses as the highest grade completed. The household level of education is defined as the highest level found among household members. Household wealth is measured by a composite indicator with five components: characteristics of the dwelling, sanitation, sources of energy, utilities, and livestock. It is measured either in absolute terms (used in this study) or as the first principal component calculated on the same items and grouped as population quintiles. Items utilized for calculating household wealth were recorded in 2001, 2003, 2007, 2011, and 2013. The household wealth status selected for the 1992–2000 events is therefore the status recorded in 2001 (29, 30).

The analysis utilized both univariate and multivariate methods. Both analyses were based on the proportion of births and deaths that were registered, as declared by the family. A small number of events with missing values (1.9% for births and 5.2% for deaths), were excluded from the final analysis (this occurs when the information concerning the recording of the event is missing). The analysis focused on time trends over the study period, age and sex differences, refugee status, level of education, and household wealth.

## Results

### Birth registration

#### Trends in birth registration

**Fig. 1:**
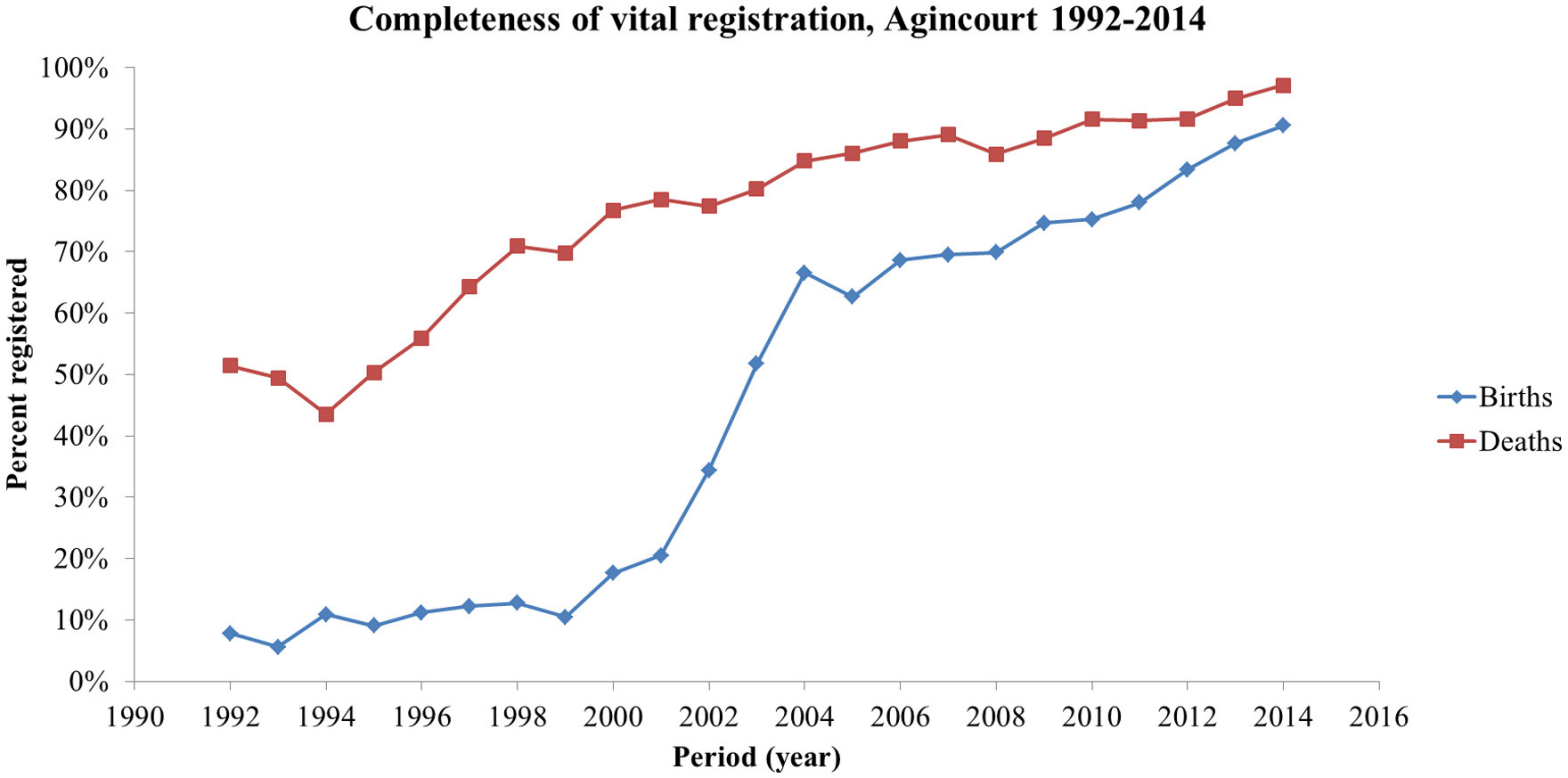
Completeness of birth and death registration, Agincourt, South Africa, 1992–2014.

A total of 42,977 births were recorded in the Agincourt HDSS from 1992 to 2014. Among those, 49.3% were registered, a value comparable to the national average for South Africa over the same period. However, the completeness of birth registration (excluding late registration) varied greatly over the years: it was only 6.2% in the first 2 years (1992–1993) and reached 89.1% in the last 2 years (2013–2014). This remarkable increase is displayed in Fig. 1. Completeness increased slowly until the year 2000, increased rapidly over the next 5 years to reach about 65%, then slowly and steadily increased to reach 90.5% in 2014. Therefore, during the course of the study, birth registration went from virtually zero (<10%) to what is considered ‘near complete’, that is, 90% and above. This remarkable achievement was also found at national level and seems unique in sub-Saharan Africa (Fig. 1).

#### Differentials in birth registration

Completeness of birth registration varied according to most factors investigated (Table 1). First, age of mother was a source of variation: the highest values of completeness were found for mid-age women, from age 22 to 36 (53.2%), and were lower for younger women (46.7% at age 17–21) and for adolescent girls (37.2% at age 12–16), as well as for older women (48.2% at age 37–41 and 37.4% at age 47–51). Lower levels among adolescents were probably due to an age effect, while lower values among older women were probably due to a cohort effect, which tended to disappear over time. Trends over time were similar for all age groups, all reaching high values of completeness in the most recent period (80.1–86.2%), with the sole exception of the adolescents (age 12–16) who were lagging behind (69.8%), although still improving very much since baseline (10.7%) (Table 2).

**Table 1:** Trends and factors of completeness of birth registration, Agincourt, South Africa, 1992–2014

		Univariate		Multivariate		
			
Variable	Value	Number of births	Percent registered (%)	Odds ratio	*p*	Significance
Period	1992–1999	12,724	10.0	1.00		Ref
	2000–2004	7,884	37.5	5.66	<10^−10^	*
	2005–2009	10,048	69.3	21.69	<10^−10^	*
	2010–2014	11,487	83.4	45.13	<10^−10^	*
Mother's age	<17	3,063	35.2	0.38	<10^−10^	*
	17–21	11,839	46.7	0.70	<10^−10^	*
	22–36	23,290	53.2	1.00		Ref
	37–41	2,918	48.2	0.88	0.0102	*
	42+	952	37.4	0.80	0.0110	*
Education	Very low	4,023	42.8	1.00		Ref
	Low	2,524	52.8	1.08	0.2046	
	Medium	3,181	65.7	1.28	<10^−4^	*
	High	4,099	76.1	1.43	<10^−8^	*
Wealth quintile	Q1	7,414	41.0	1.00		Ref
	Q2	7,535	47.7	1.28	<10^−8^	*
	Q3	7,542	49.6	1.35	<10^−10^	*
	Q4	7,264	51.0	1.51	<10^−10^	*
	Q5	6,947	53.7	1.81	<10^−10^	*
Refugee status	South African	27,173	53.0	1.00		Ref
	Mozambican	14,881	42.6	0.72	<10^−10^	*
Sex of child	Female	21,058	49.0	1.00		Ref
	Male	21,080	49.5	1.02	0.4186	
	Total	42,143	49.3			

* *p*<0.05; Ref=reference category for multivariate analysis.

**Table 2:** Interaction between factors of completeness of birth registration and period, Agincourt, South Africa

	Percent of births registered, by period				
	
Factor	1992–1994 (%)	1995–1999 (%)	2000–2004 (%)	2005–2009 (%)	2010–2014 (%)
Age of mother					
12–16	10.7	5.6	21.4	47.8	69.8
17–21	7.1	9.1	32.7	67.1	80.8
22–36	8.4	13.0	42.3	73.3	86.2
37–41	6.7	11.8	39.8	70.5	83.0
42–51	6.6	7.9	35.7	71.4	80.1
Refugee status					
Mozambican	3.6	6.3	32.3	66.3	76.8
South African	10.7	14.1	40.4	70.9	86.8
Level of education					
Very low	7.7	9.7	29.0	68.3	81.0
Low	8.3	8.7	30.2	69.9	82.4
Medium	12.4	11.8	36.1	73.0	85.9
High	20.0	11.5	39.3	70.4	90.3
Household wealth					
Lowest	5.4	7.3	22.1	53.0	66.5
Low	3.7	5.1	28.4	59.7	73.5
Medium	7.1	8.9	36.4	67.8	79.9
High	9.2	12.6	40.1	70.7	84.7
Highest	14.4	19.4	45.5	74.0	88.5

There was no difference in birth registration by sex of the child, neither at baseline nor at end point.

Mozambican refugees had somewhat lower levels of completeness of birth registration, on average 42.6% compared to 53.0% for South Africans. Trends in completeness were very much parallel for both groups, with a regular gap of about 10% over the years, so that in the most recent period (2010–2014) completeness had reached 76.8% among Mozambicans for 86.8% among South Africans.

Gradients in completeness by socioeconomic status were as expected, with higher values for households with higher levels of education and wealth. Completeness varied from 42.8% on average for households with a very low level of education (less than Grade 7) to 76.1% for households with higher levels (completed high school, ‘matric’ and above). Here again, the gap between the high and low levels stayed approximately the same (about 10%) over the years, so that in the most recent period (2010–2014) completeness was high for all groups, ranging from 81.0% for a very low level of education to 90.3% for a high level of education.

Gradients by wealth were also consistent but with a small interaction with period. Completeness was uniformly low at baseline (1992–1994) for all wealth groups, ranging from 5.4% for the poorest to 14.4% for the wealthiest, whereas completeness was much higher for all wealth groups in the most recent period (2010–2014), ranging from 66.5% for the poorest to 88.5% for the wealthiest. The gap between the two extremes tended to increase overtime, from 9 to 22%, which means that improvements for the poorest groups were somewhat slower than for the other groups, although still important given the situation at baseline.

#### Multivariate analysis

The multivariate analysis confirmed the observations of the univariate analysis: all factors investigated were highly statistically significant, except for the sex of the child. All factors appeared largely independent from each other, because the gradients found in the multivariate analysis were basically the same as those identified in the univariate analysis. In terms of the odds ratio (OR) associated with completeness of birth registration, the largest values (positive or negative) were found for period (OR=45.1 for 2010–2014) and to a lesser extent for age (OR=0.38 for age 12–17) and wealth (OR=1.81 for the wealthiest), and the lowest values were found for refugee status (OR=0.72) and level of education (OR=1.43 for the highest level of education) (Table 1).

### Death registration

#### Trends in death registration

The situation for death registration was quite different from that for birth registration (Table 3). First, the completeness of death registration was much higher (82.4%) than that for births over the whole study period. Second, it was also much higher at baseline (1992–1994): 47.5%, compared with 8.0% for births. Progression over time was also steady and impressive, reaching high values (93.4%) in the most recent period (2010–2014) and 97% in 2014 (see Fig. 1). This major improvement in Agincourt seems to match that found at national level.

**Table 3:** Trends and factors of completeness of death registration, Agincourt, South Africa, 1992–2014

		Univariate		Multivariate		
			
Variable	Value	Number of births	Percent registered (%)	Odds ratio	*p*	Significance
Period	1992–1994	689	47.5	1.00		Ref
	1995–1999	1,733	63.0	2.07	<10^−10^	*
	2000–2004	3,155	79.8	7.08	<10^−10^	*
	2005–2009	4,398	87.5	14.64	<10^−10^	*
	2010–2014	3,903	93.4	31.80	<10^−10^	*
Age at death	0–4	2,049	33.7	0.03	<10^−10^	*
	5–9	201	78.1	0.31	<10^−7^	*
	10–19	433	83.4	0.53	<10^−4^	*
	20–39	3,801	92.9	1.00		Ref
	40–59	3,262	92.5	1.03	0.0792	
	60–79	2,786	88.4	0.78	0.0108	*
	80–99	1,346	89.9	0.68	0.0014	*
Education	Very low	1,543	77.9	1.00		Ref
	Low	1,152	79.3	0.97	0.818	
	Medium	1,608	81.8	1.05	0.714	
	High	2,397	83.9	1.17	0.174	
Wealth quintile	Q1	3,215	75.9	1.00		Ref
	Q2	2,494	81.8	1.55	<10^−6^	*
	Q3	2,276	84.6	1.82	<10^−8^	*
	Q4	2,195	87.5	2.18	<10^−10^	*
	Q5	1,906	90.1	2.57	<10^−10^	*
Refugee status	South African	9,696	87.4	1.00		Ref
	Mozambican	4,154	70.8	0.30	<10^−10^	*
Sex of child	Female	6,592	82.4	1.00		Ref
	Male	7,280	82.4	1.04	0.539	
	Total	13,878	82.4			

* *p*<0.05; Ref =reference category for multivariate analysis.

#### Differentials in death registration

Gradients by age at death were more pronounced than gradients by age of mother for births. In particular, completeness was uniformly high for adults (91.3% for age 20–99) and abnormally low for under-five children (33.7%), and particularly for infants (26.7%), whereas it reached higher values for older children (78.1% at age 5–9 and 83.4% at age 10–19). The rise in completeness by age, from birth to age 20, was steady and rapid from age 0 (26.7%) to age 5 (81.3%). This situation is surprising and could lead to major confusion if analysis were based on registered deaths of children. However, it seems peculiar to this area or at least much more pronounced than at national level (Table 4).

**Table 4:** Interaction between factors of completeness of death registration and period, Agincourt, South Africa

	Percent of deaths registered, by period				
	
Factor	1992–1994 (%)	1995–1999 (%)	2000–2004 (%)	2005–2009 (%)	2010–2014 (%)
Age at death					
0–4	12.3	24.2	23.9	41.3	51.0
5–9	38.5	31.3	85.4	90.3	96.2
10–19	41.0	64.6	86.0	90.6	99.1
20–39	56.3	75.4	91.8	96.3	98.6
40–59	57.3	77.8	91.8	96.1	98.3
60–79	64.9	69.8	92.0	93.6	97.8
80–99	59.1	70.9	86.7	92.7	98.7
Refugee status					
Mozambican	19.3	37.2	64.7	78.0	89.0
South African	58.0	72.4	86.5	92.0	95.3
Level of education					
Very low	54.2	56.4	70.9	83.5	91.7
Low	47.5	58.6	76.0	85.7	91.3
Medium	38.2	59.7	82.1	86.0	91.6
High	57.4	70.2	81.4	87.1	91.4
Household wealth					
Lowest	38.9	39.7	61.6	78.4	85.0
Low	40.5	59.4	73.2	81.1	86.4
Medium	50.7	56.3	77.5	86.6	93.2
High	56.6	70.7	85.1	89.5	94.3
Highest	59.4	80.4	90.7	93.0	97.8

As for births, there was no variation by sex, both males and females being equally registered (82.4%). There was no interaction with age at death, even for children under five, nor with period, with the sole exception of the 1992–1994 period when males were somewhat better registered than females (*p*<0.001). Completeness of death registration after the year 2000 was slightly higher for females (87.9%) than for males (87.0%), but the difference was not statistically significant (*p*=0.152).

With respect to age, increases in completeness were marked in all age groups (+46% on average). The age pattern of completeness was somewhat different at baseline, with increasing values with increasing age, from 12.3% at age 0–4 to 64.9% at age 60–79. At end point (2010–2014), completeness was uniformly high (>96%) for all age groups except for children under five, where it remained average (51%). As a consequence, the largest gains were for younger age groups (age 5–59), aside from the under-five children. However, even for the under-five children progress was remarkable, with a marked absolute increase of +39%, because it started from very low values.

Mozambican refugees started with very low values of death registration at baseline (19.3%), way below South Africans (58.0%), but recovered quickly, reaching 89% in the most recent period, not far from South Africans (95.3%).

Level of education was a minor factor in completeness of death registration at baseline and had almost disappeared at end point, since all categories were above 90% in 2010–2014. As for birth registration, gradients by level of education were rather small over the whole period.

Progress was steady and impressive for all wealth categories, as was the case for birth registration. At baseline, the gradient by wealth was marked, from 38.9% at the lowest level of wealth to 59.4% at the highest level. At end point, the differences were smaller and the most advanced groups were all above 93%, with the lowest group at 85%. As a consequence, the difference between the highest and lowest wealth groups was about halved.

#### Multivariate analysis

As for birth registration, the multivariate analysis of completeness of death registration confirmed the univariate analysis, most differences being highly significant, with the exceptions of sex of the deceased person and of education. Gradients were also similar, showing the large independence between all explanatory variables (Table 3). The largest odds ratios (OR=31.8) were found for the period 2010–2014. Some age groups also had very low odds ratios, especially among children (OR=0.03 for the age group 0–4; OR=0.31 for the age group 5–9; OR=0.53 for the age group 10–19). The multivariate analysis showed significant differences for older persons (OR=0.78 at age 60–79 and OR=0.68 at age 80–99), which came mostly from the interaction between age and period, as older age groups did better in the early part of the study compared with younger adults. The odds ratio was also low for Mozambicans (OR=0.30) and lower than for birth registration. However, as seen above, the differences between refugees and South Africans were largely reduced in recent years. Gradients by level of education were so small that they were not statistically significant. In contrast, gradients by household wealth were marked, with odds ratio reaching 2.57 for the highest quintile (Table 3).

## Discussion

In the Agincourt population, the improvements in vital registration were pervasive. In the Agincourt HDSS area, as it is the case for South Africa as a whole, the completeness of vital events (births and deaths) increased markedly after the new law passed in 1992 and as a result of the strong political will of the new government elected in 1994. In remote areas, and in particular in the former homelands, birth registration was virtually non-existent before 1994 yet was near completion 20 years later. Likewise, death registration was deficient before 1994 but reached high levels in recent years. This major achievement is a real ‘success story’ for African countries.

The improvement in completeness of vital registration is due to the major concerted efforts of the South African government to register all births and deaths for all population groups, including in remote areas. This was achieved by reorganizing the CRVS system to accommodate the whole population, by developing a full-scale population register with a single ID number, by developing the infrastructure for registration (fixed points and mobile teams), by closely involving the hospitals and clinics where these events occur, by developing a fully computerized system with Internet connections, and by large-scale information and advocacy campaigns in the whole country. This new situation will enable the political and public health authorities, as well as researchers and concerned persons, to have a better understanding of the rapidly changing trends in fertility and mortality, two crucial components of population dynamics (37).

The registration of births and deaths was facilitated by the fact that certificates are now needed for many procedures, in particular the fact that birth certificates are needed for ID cards (necessary for voting, to obtain a driver's license, and for many other purposes) and for school enrollment, and that death certificates are required for burials in cemeteries and for accessing pensions for widows or widowers. Numerous changes have also occurred in South Africa over this period, some of which could have an impact on birth and death registration, in particular the strong incentives for getting access to social grants. Since the late 1990s, the country has developed a generous system of social grants for children, orphans, the elderly, and handicapped persons, all requiring an ID, a birth certificate, or a death certificate depending on the case. For example, the child support grant is a strong incentive to mothers to have their children registered; the foster child grant is an incentive to register the deaths of persons who left behind orphans; and the older person grant requires an ID card, as do the disability grant and the care dependency grant (37).

Another feature contributing to the success of CRVS in South Africa is the high level of development of the country. A high gross domestic product and an efficient tax system are necessary for financing the infrastructure and the functioning of the CRVS system, as well as for supporting the social grants. Last, the high level of education of the whole population, males and females alike, the relatively high level of urbanization, and the strong computer and Internet infrastructure all contributed to the achievement (37).

Turning to the present study, the improvements were pervasive in all demographic and socioeconomic groups. They reached all age groups, both sexes alike, all population strata, all socioeconomic status groups. Groups that started from very low vital registration values remain under the threshold of 90% completeness, but these groups are still moving up, and if they lag behind, it is only by a small margin, equivalent to a few years of improvements.

A few problematic groups for the recent period are worth noting. For birth registration, in relative terms, the main issues were among the births to adolescents, a problematic group for many reasons (38); the very poor households; and to a lesser extent the Mozambican refugees. For death registration, also in relative terms, the main issues were among children under five, particularly infant deaths, and to a lesser extent among the very poor households and the Mozambican refugees.

In absolute terms, the situation was somewhat different: socioeconomic status mattered little, and age was the main factor in lack of registration in recent years. Among births that occurred in 2013–2014, 40% of the unregistered cases (*n*=549) were among women less than 22 years of age. Among the deaths that occurred in 2013–2014, 79% of the unregistered cases (*n*=63) were among under-five children. These age groups should be targeted for improving completeness in the future.

The difference between birth and death registration has almost disappeared now that completeness is high. However, this was not the case before 1994, when there were major differences between these events. This difference could have been due to a variety of factors, in particular to the needs for people to register either birth or death. In particular, a death certificate was needed earlier for adults to be buried in a cemetery, which could explain the higher coverage for death registration before 1994. These differences could be further investigated retrospectively.

Despite being very specific in its ethnic composition and geographical location, the population of Agincourt usually fares close to the national average in terms of demographic and socioeconomic indicators (fertility, mortality, nuptiality, education, wealth, etc.). This seems to be the case also for completeness of vital registration. There is no precise trend data at national level to compare with Agincourt, but the available evidence goes in the same direction. There are a few differences, however, probably due to the fact that Agincourt is fully rural and was part of the homeland system earlier on. For births, the very low values of completeness at onset (8% in Agincourt compared to about 21% at national level) lasted for about 10 years, and it took another 10 years to fill the gap, so that after 2010 completeness in Agincourt had reached the national level. For deaths, coverage also seemed somewhat lower at onset (46% versus 56% at national level) and was apparently somewhat higher at end point, although precise data are lacking at national level. This could be an indirect effect of the HDSS and especially of the comprehensive investigation of deaths by verbal autopsy, which has been going on since 1992 (39).

The issue of recording causes of death was not addressed in this study. Agincourt has maintained a comprehensive investigation of causes by verbal autopsy for all deaths that occurred in the study area. Adding verbal autopsies to death registration has been proposed for improving health information systems in areas where medical certification of causes of death is lacking (40).

The Agincourt study relied on family declaration of the registration of births and deaths. There is no reason to doubt the quality of this information, as families receive an official certificate and are well aware of their rights and duties. In an ideal world, one would like to match the HDSS information with the official records, event by event. An attempt to do so for the deaths that occurred in 1992–1995 showed how difficult such a task would be. Out of the 1,001 deaths recorded in the population, only 187 could be matched by name in hospital registers (38). Another recent attempt conducted in 2006–2009 showed that there was no major bias in death registration between HDSS and CRVS in the Agincourt area, demonstrated that 60.8% of death records could be matched using complex procedures, and confirmed that those that could not be matched were mostly the deaths of young children, those in poorer households, and Mozambicans, most likely because these deaths did not occur in hospitals and were never registered (41).

A large part of the gap in birth registration was compensated for recently by late registration nationwide. The Agincourt study was not designed to cover this issue, but more investigation could be done in the future to investigate whether full birth registration is achieved before children enter school, whether on time or late.

Although major improvements have occurred since 1994 and trends remain favorable, efforts should continue in the future to achieve full completeness in birth and death registration, as in more developed countries. Targeting the age groups where the most gaps were found could help in attaining this goal.

## Paper context

Vital registration remains limited in Africa. Estimation of completeness of birth and death registration is hazardous because of lack of precision on the total number of events in national populations. This study reports on a health and demographic surveillance system located in a rural community of South Africa (Agincourt). It shows that birth and death registration increased from low to high values within a 20-year period, a major improvement in a short period of time.
